# Large-Scale Wireless Temperature Monitoring System for Liquefied Petroleum Gas Storage Tanks

**DOI:** 10.3390/s150923745

**Published:** 2015-09-18

**Authors:** Guangwen Fan, Yu Shen, Xiaowei Hao, Zongming Yuan, Zhi Zhou

**Affiliations:** 1 School of Petroleum Engineering, Southwest Petroleum University, Chengdu 610500, China; E-Mail: zmyuan028@163.com; 2 School of Civil Engineering, Dalian University of Technology, Dalian 116024, China; E-Mails: shenyu@mail.dlut.edu.cn (Y.S.); hao123@mail.dlut.edu.cn (X.H.); zhouzhi@dlut.edu.cn (Z.Z.)

**Keywords:** wireless sensors, temperature, liquefied petroleum gas storage tank, structural health monitoring technology

## Abstract

Temperature distribution is a critical indicator of the health condition for Liquefied Petroleum Gas (LPG) storage tanks. In this paper, we present a large-scale wireless temperature monitoring system to evaluate the safety of LPG storage tanks. The system includes wireless sensors networks, high temperature fiber-optic sensors, and monitoring software. Finally, a case study on real-world LPG storage tanks proves the feasibility of the system. The unique features of wireless transmission, automatic data acquisition and management, local and remote access make the developed system a good alternative for temperature monitoring of LPG storage tanks in practical applications.

## 1. Introduction

The demand for energy resources increased sharply over the past decades in China. In 2014, it was estimated that China consumed 20% of the global energy [1], ranking the first in the world. The growth rate of energy consumption in China will maintain an average speed of 4.5% in the next 20 years [2], which will result in the consumption of 82 billion tons of coal, 12 billion tons of crude oil, and 5.8 trillion of natural gas.

Among various kinds of energy resources, the Liquefied Petroleum Gas (LPG) is recognized for its efficiency, environmental friendliness, and convenience. These unique features make LPG an ideal choice for rural heating, motor fuel, refrigeration, and cooking. However, LPG is a dangerous chemical which is highly flammable and explosive [3]. Every year, accidents related to LPG storage tanks lead to tremendous property and life losses [4]. For example, in 2009, an oil storage tank explosion in West India caused 13 deaths and more than 150 wounded; in 2013, an oil-transporting vehicle exploded in Democratic Republic of the Congo, leading to 242 deaths; and on 2 April 2013, an explosion of an oil storage tank in Dalian, China, caused two missing people and two badly injured. Since the accidents of LPG storage tank explosion significantly threat the lives of people, the importance to ensure the safe operation of the LPG storage tanks through monitoring technology is widely acknowledged.

Among various monitoring parameters such as temperature, strain, pressure, refractive index, displacement, vibration, *etc.*, temperature is one of the most critical factors to evaluate the safe status of the LPG storage tanks [5,6]. In recent years, as an alternative to manual surveying, various temperature sensors, and sensing systems for LPG storage tank monitoring has been investigated [7,8,9,10,11]. Yiwei *et al.* designed a real-time distributed measurement system based on field bus technology for large-scale oil tanks. The system utilized optical fiber sensors to collect the oil tank’s data and Ethernet to achieve long-distance data transmission by RS-485. Together with a computer to collect and analyze the data, monitoring and managing oil tanks is achieved intelligently [12]. Liu *et al.* proposed to use fiber Bragg grating sensors to conduct real-time measurements of the temperature and pressure in the gas tanks [13]. Lei proposed a multi-point temperature monitoring system based on infrared thermal imagers for the Liquefied Nature Gas (LNG) storage tank monitoring [14]. However, the long-term engineering validations reveal some shortcomings of the above wired monitoring systems such as high complexity for the field deployment of the monitoring system and potential new hazards in tank operations. As a result, the wired technology cannot meet the challenges of health monitoring for the LPG storage tanks.

To solve the challenges of insufficiency of the current temperature monitoring systems, this paper developed an integrated monitoring system which combines wireless temperature sensors, fiber optical high-temperature sensors, a large scale sensor network, and a management software to sense, collect, store, display, and manage the monitored data. In Section 2, hardware for the monitoring system was designed and implemented followed by software in Section 3. In Section 4, the developed large-scale wireless temperature monitoring system was applied on a LPG storage tank to further test and validate the feasibility of the system.

## 2. Hardware Design and Implementation

**Figure 1:**
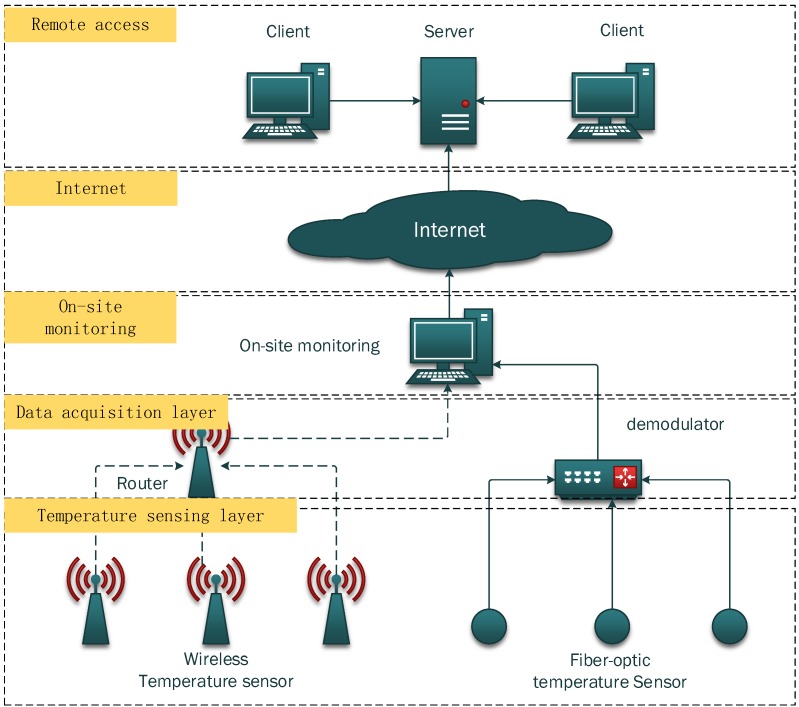
Hardware design for the monitoring system.

Figure 1 shows the hardware design for the wireless temperature monitoring system. The hardware consists of four layers. From bottom to top, these layers are temperature sensing layer, data acquisition layer, on-site monitoring layer, and the remote access layer.

The temperature sensing layer is responsible to measure the raw temperature data from the LPG storage tanks. In this layer, both wireless temperature sensors and fiber optic temperature sensors are utilized. The wireless temperature sensors are easy to deploy while the fiber optic sensor is free of electric shock and can be deployed in certain critical areas. The data acquisition layer is to collect the data from the temperature sensing layer. This layer has routers to receive data from the wireless temperature sensors and demodulators to obtain data from the fiber optic sensors. The demodulator is an OSA with MATLAB programs inside. The programs can calculate the L parameter of the FP cavity and further to get the temperature data by using cavity length *vs.* temperature function we will discuss in Section 2.2. The routers deployed in the fields cover a wide range of area, enabling a large number of wireless temperature sensors to monitor the LPG storage tanks. The on-site monitoring layer, therefore, collects data from routers and demodulators. The on-site monitoring system gets the data from the routers via a wireless receiver and from the demodulator through a LAN cable. With the collected data, the on-site monitoring server displays all the temperature information via monitoring software, which will be further discussed in next section. In addition to data display, the on-site monitoring layer also forwards all the monitored temperature data to the remote server allowing remote access from the clients, which is the top layer of this system. The remote access layer has one server and several clients. The server receives the temperature data obtained from several different on-site monitoring servers, store, and analyze the temperature information in the database. The server also provide web services for the clients, allowing people to have access to the temperature information from everywhere in the world. Details for all the layers are discussed in following subsections.

### 2.1. Wireless Sensor and Sensor Network

**Figure 2:**
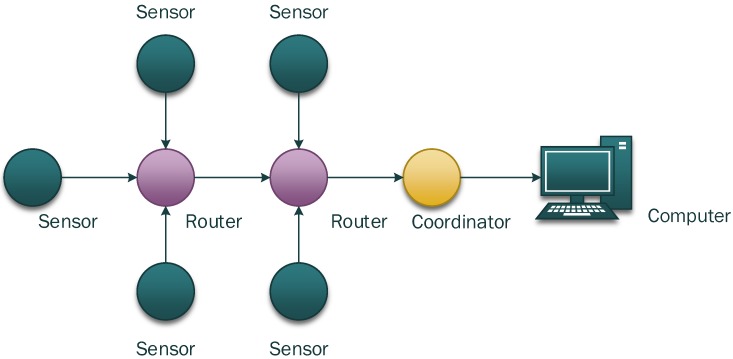
Framework for ZigBee wireless sensor network.

**Figure 3:**
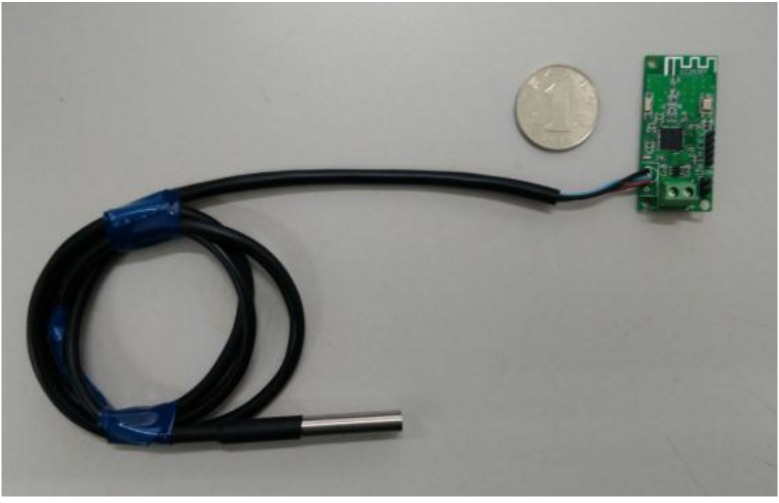
Wireless sensing node.

**Figure 4:**
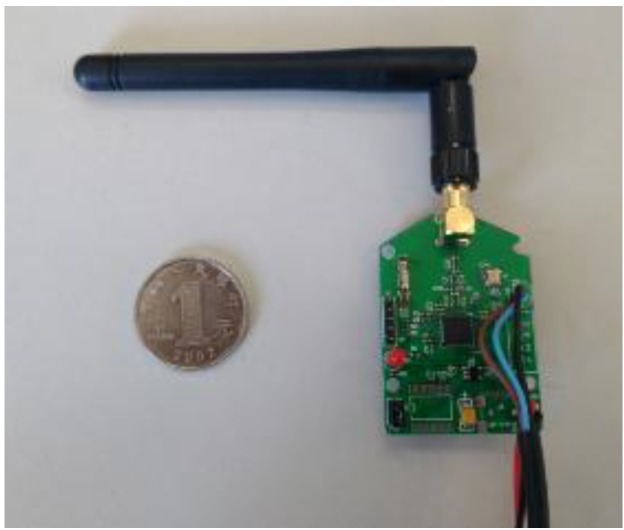
Router.

**Figure 5:**
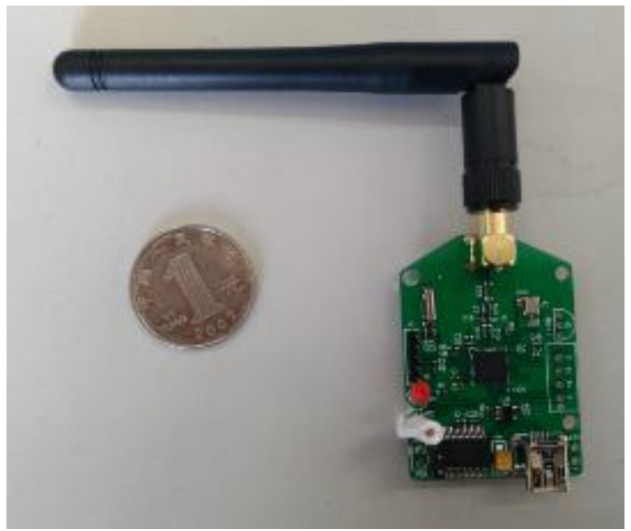
Receiver.

A Zigbee wireless sensor network is used in this study. Figure 2 shows the framework of the applied network, which consist of wireless temperature sensing nodes, routers and receivers (also known as coordinator). The wireless temperature sensing node, as shown in Figure 3, measures the raw temperature and sends the data to the router. The router, as shown in Figure 4, then forwards the data to the wireless receiver, as shown in Figure 5. A plurality of routers operate in multi-hop transmission; hence, the wireless signal is able to cover a wide range of area.

As shown in Figure 3, a digital temperature sensor (model DS18B20) and a system-on-chip solution (SoC chip CC2530) were utilized to form the wireless sensing network. The DS18020 digital temperature sensor has a unique one-wire interface which requires only one port pin for communication. It has a temperature measurement range from −55 to +125 °C and an accuracy of ±0.5 °C, which make it an excellent choice for the temperature monitoring. The CC2530 chip is an IEEE 802.15.4 compliant true system-on chip, supporting the proprietary 802.15.4 market as well as the ZigBee standards. The CC2530 offers a 101 dB link budget, which has an excellent receiver sensitivity and robustness to interference, four power modes, multiple FLASH sizes, and extensive peripheral set including two USARTs, 12Bit ADC, and 21 general-purpose GPIO, as well as an industry-standard enhanced 8051 MCU core.

**Figure 6:**
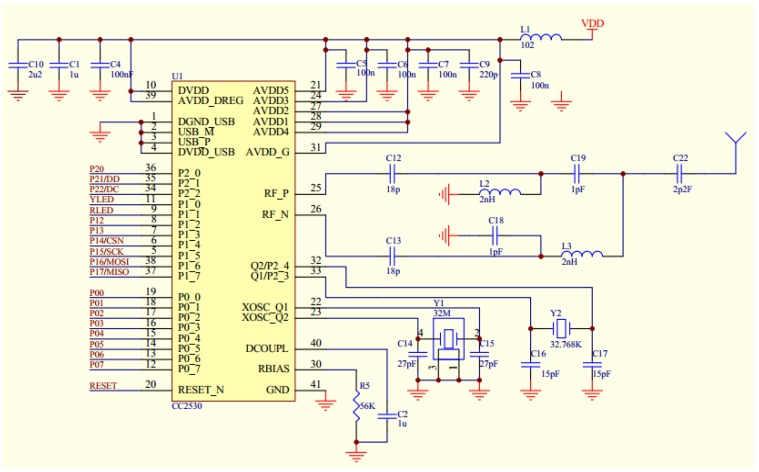

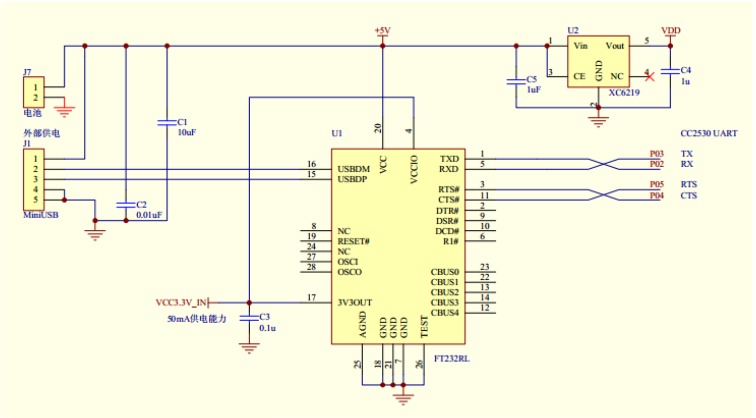
Schematic design of PCB.

The excellent functionalities provided by the DS18B20 and the CC2530 make it possible to achieve a unified design. Schematic design of the Printed Circuit Board for the wireless temperature sensing node, router and receiver are outlined in Figure 6. In the design of the wireless temperature sensing node, an onboard PCB antenna was utilized in order to make the sensing node to be compact in size. In the design of the router and receiver, an external antenna was utilized to achieve high signal to noise ratio.

The performances of hardware were tested and Table 1 shows the tested results. The temperature measurement range is 0 °C~+125 °C which meet the basic temperature monitoring requirements for the LPG storage tanks. The measurement resolution is 0.1 °C and the measurement precision is ±0.5 °C. The data acquisition interval ranges from one second to 24 h and the default set up is 10 min. The wireless transmission range is 50 to 300 m and can be extended by adding more routers. The working frequency is 2.4 GHz and the system power voltage is 3.0–5.5 VDC, which can be powered by battery or by solar energy based on the users’ needs. The system has a relative low energy consumption, which is mainly due to the onboard sleep mode. In the normal operation mode the system has a working current of 10 mA, while in the sleep mode the current drops to 10 µA. The normal operation mode activates only when the data acquisition interval is arrive. The sleep mode activates by the onboard software when the data transmission completed. Considering the transmission rate of the ZigBee system, the data transmission is done in less than 5 ms. Thus, most of the time the system is working in the sleep mode. Based on the experiment data and the on-site experience a single sensor node powered by a typical 2000 mAh lithium battery has a life of more than two years working at a data acquisition interval of two minutes.

**Table 1:** Performances of hardware

Item	Performances
Temperature Range	0 °C~+125 °C
Resolution	0.1 °C
Precision	±0.5 °C
Data acquisition interval	1 s~24 h
Wireless transmission range	50~300 m
Max sensing nodes	65,535
Working frequency	2.4 GHz
System power voltage	3.0~5.5 V/DC

### 2.2. Fiber Optic Temperature Sensor

Fiber optic sensor has several advantages over conventional sensors: immunity to electromagnetic interference, ability to operate in harsh environments and potential for multiplexing. It has high sensitivity and is widely used in the demanding measurements of various physical quantities, such as temperature, strain, pressure, refractive index, displacement, vibration, *etc*. It is an excellent candidate to use on the oil storage tank where electric charge and discharge are strictly prohibited as even a tiny spark can light the flammable gas and causing disasters. Therefore, the application of fiber optic sensors not only allow for high precision but also minimize the risk of fire hazard caused by monitoring system.

**Figure 7:**
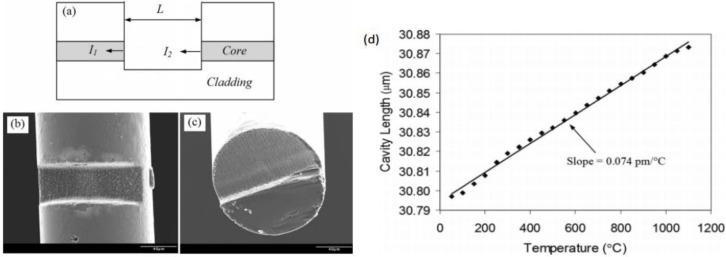
Schematic structure and SEM images of fiber inline FPI device fabricated by femtosecond laser ablation and corresponding response to temperature variation. (**a**) Structural illustration; (**b**) Top view; (**c**) Cross-section; (**d**) Temp. *vs.* Cavity Length.

In this study, we used a miniaturized fiber inline FP interferometer temperature sensor fabricated by femtosecond laser. As shown in Figure 7a–c, an open cavity was formed by one-step micromachining a micro notch in a single-mode optical fiber using a femtosecond laser. The device has an all-glass structure and does not involve the assembly of multiple components. As a result, we expect that the device will survive very high temperatures which make it suitable for temperature monitoring in the oil storage tank.

The device fabrication was carried out using a home-integrated fs laser 3D micromachining system. The repetition rate, center wavelength, and pulse width of the femotosecond (fs) laser (Legend-F, Coherent, Inc. Santa Clara, CA, USA) were 1 kHz, 800 nm, and 120 fs, respectively. The maximum output power of the fs laser was approximately 1 W. We used the combination of waveplates and polarizers to reduce the laser power to about 20 mW, and then used several neutral density (ND) filters to further reduce the laser power to desirable values. The attenuated laser beam was directed into an objective lens (Olympus UMPLFL 20X) with a numerical aperture (NA) of 0.45 and focused onto the single mode optical fiber (Corning SMF 28) mounted on a computer-controlled five-axis translation stage (Aerotech, Inc. Pittsburgh, PA, United States) with a resolution of 1 mm.

During fabrication, the interference signal of the fiber FP device was continuously monitored. A tunable laser source (HP 8168E) was connected to one of the input ports of the 3 dB fiber coupler. The output port of the coupler was connected to the device under fabrication. Controlled by the computer, the tunable laser continuously scanned through its wavelength range (1475–1575 nm) at the rate of 1 nm per step. The signal reflected from the device at each wavelength step was recorded by an optical power meter (Agilent 8163A). The fabrication was stopped after a well-formed interference pattern was recorded. The interference signal is interrogated based on the two-beam optical interference equation as follows:

(1) $$I=I_{1}+I_{2}+2\sqrt{I_{1}I_{2}}\cos(\frac{4\text{π}L}{\text{λ}}+{\text{Φ}}_{0})$$

where I is the intensity of the interference signal, $I_{1}$ and $I_{2}$ are the reflections at the cavity surfaces, respectively, $\Phi_{0}$ is the initial phase of the interference, L is the optical length of the cavity, and λ is the optical wavelength. According to Equation (2), the two adjacent interference minimums have a phase difference of 2π. That is

(2) $$\left(\frac{4\text{π}L}{{\text{λ}}_{1}}+{\text{φ}}_{0}\right)-\left(\frac{4\text{π}L}{{\text{λ}}_{2}}+{\text{φ}}_{0}\right)=2\text{π}$$

where λ_1_ and λ_2_ are the wavelengths of two adjacent valleys (Figure 3) in the interference spectrum. The optical length of the FP cavity can thus be found as $L=0.5{\text{λ}}_{1}{\text{λ}}_{2}/({\text{λ}}_{2}-{\text{λ}}_{1})$. Based on the interference spectrum we obtained from the optical power meter (Agilent 8163A) in the fabrication process, the two neighboring valleys are 1489.1 nm and 1525.1 nm, we calculated that the FP cavity length was 30.797 μm, which was very close to the length estimated by SEM imaging.

The fabricated device was tested for its ability to survive high temperatures. The sensor was placed horizontally in a quartz tube with one-inch inner diameter, hosted inside a programmable electric tubular furnace. The temperature of the furnace was increased from room 50 °C to 1100 °C (limited by the highest temperature of the furnace used) at a step of 50 °C and the interference spectra at these temperatures were recorded. However, to improve the accuracy, we replaced the tunable laser with a broadband source made by multiplexing a C-band and an L-band Erbium doped fiber ASE (amplified spontaneous emission) sources. The interference fringe reflected from the device was recorded by an optical spectrum analyzer (OSA, HP 70952B).

The cavity length as a function of the temperature is plotted in Figure 1d, where it increased nearly linearly following the increase of temperature. The fiber FP device successfully survived high temperatures up to 1100 °C. Even if not lasting long at the very high temperature, it is good enough to meet the needs of early warning of the LPG storage tank fire hazards. The temperature sensitivity of this particular FP device was estimated to be 0.074 pm/°C based on the linear fit of the measurement data. The equivalent CTE of the device was calculated to be 2.4 × 10^−6^/°C, which was about four times larger than the known CTE of the fiber cladding (fused silica, 5.5 × 10^−7^/°C). The thermal test was repeated several times and the results were quite reproducible.

**Figure 8:**
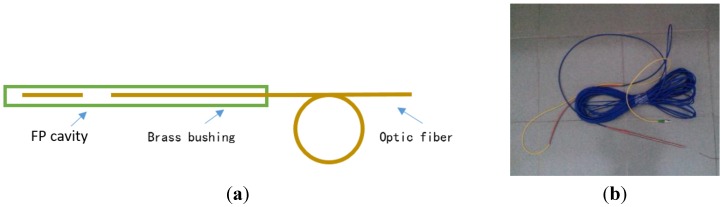
Schematic design and photo of the actual sensor. (**a**) Schematic design of fiber optical temperature sensor; (**b**) The actual temperature sensor.

For the protection of the miniaturized fiber inline FP interferometer temperature sensor, a brass bushing packaging technique was introduced. Figure 8a shows the schematic design of the packaged sensor and Figure 8b shows the actual packaged temperature sensor.

### 2.3. Onsite Monitoring System

Onsite monitoring system receives data from the sensing networks and displays the data via the LPG storage tank monitoring software. The onsite monitoring system consists of wireless receivers (as mentioned in the previous section), a demodulator, a computer, a network module, and an Uninterrupted Power Supply system (UPS). The wireless receiver receives temperature information from the wireless sensing node and sends configuration command to the wireless sensing node. The demodulator collects of data from the fiber optical sensors. The computer can store and display the monitored data received. In case of power failure, a UPS is introduced to grantee a reliable power source.

### 2.4. Remote Access System

**Figure 9:**
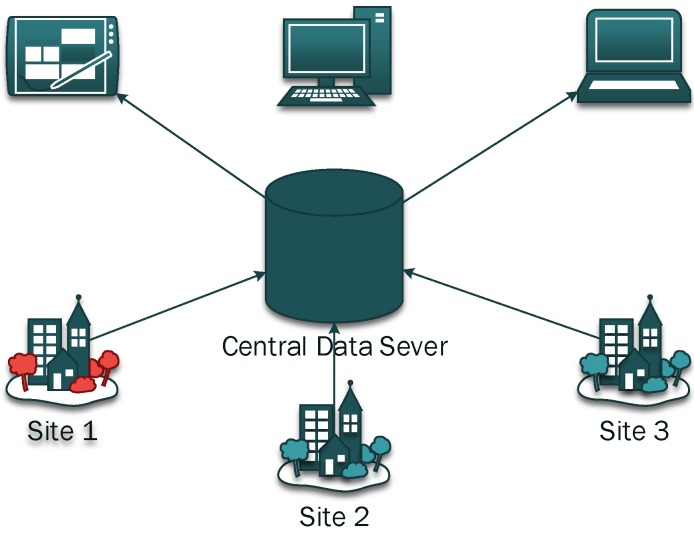
Client/Server framework.

Client/Server framework is desirable to implement the idea of centralized storage and distributed sensing. It ensures a plurality of onsite monitoring system that is currently in monitoring can submit all the data collected. The monitoring personnel can get access to any one of the onsite monitoring system in remote. Figure 9 shows the C/S framework.

**Figure 10:**
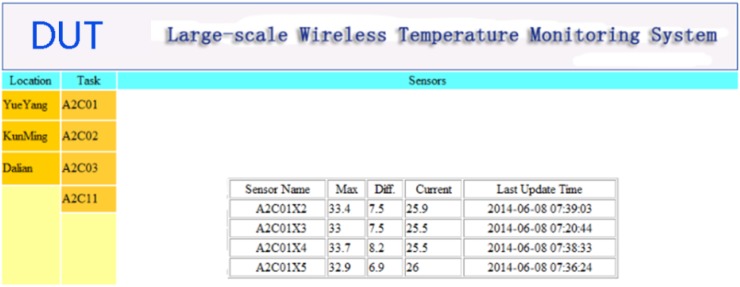
Remote access.

The C/S framework consists of multiple onsite monitoring systems (as in the previous section), a central data server and remote access terminals. The central data server has a typical LAMP architecture, in which L stands for Linux, A stands for Apache, M stands for MySQL, and P stands for PHP. The MySQL is the world’s second most widely used relational database management system (RDBMS) and most widely used open-source RDBMS. The Apache HTTP Server, colloquially called Apache is the world’s most widely used web server software. The PHP is a programming language for the writing of active web pages. Together with Linux, apache, MySQL and PHP guarantee an easy approach to ensure remote access via any device connected to the Internet, e.g., Notebook, PC, as shown in Figure 9. Figure 10 shows an example interface of the homepage for the monitoring web site.

## 3. Software Design and Implementation

**Figure 11:**
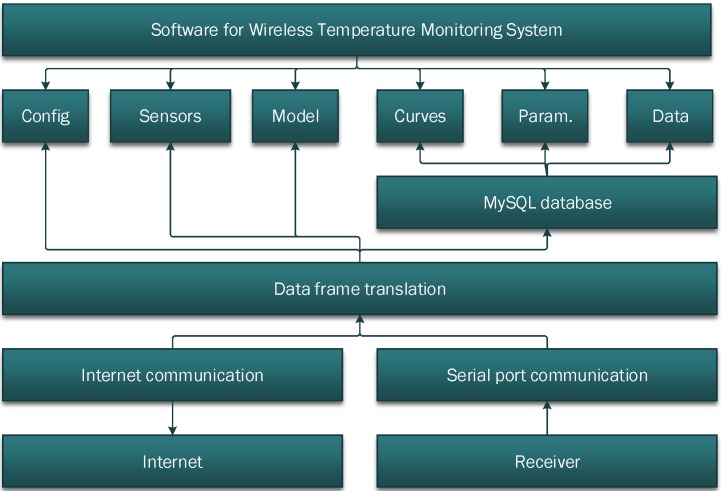
Software modules.

The monitoring software stores and displays the monitoring parameters from the sensing networks such as the in-tank temperature, tank surface temperature, and the environment temperature. In addition, the software has a build-in function to automatically calculate the temperature differentials between the tank surface and the inner of the tank, the temperature changing rate, and the max history temperature. The software also manages the data according to the monitoring tasks and can export to Excel files for further processing. The software has six modules as shown in Figure 11 including configuration (config.), sensors, model, curves, parameter (param.), and data modules, which will be discussed in detail in following subsections.

### 3.1. Configuration Module

**Figure 12:**
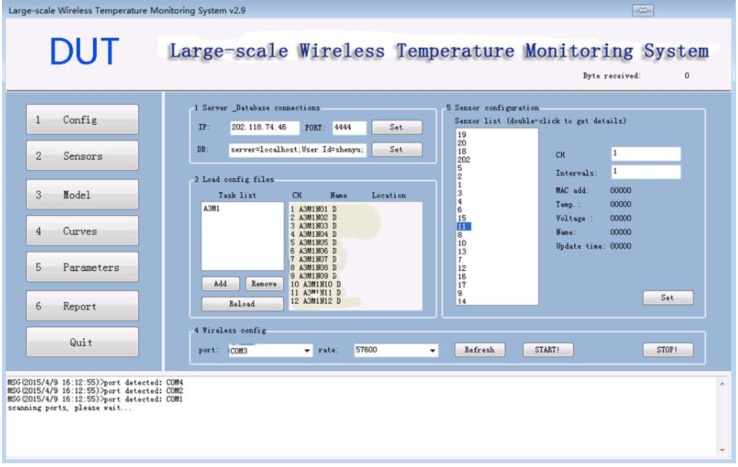
Configuration Module.

The configuration module as shown in Figure 12 has four sub-modules, the database path and remote server address module, the task module, the communication port number, and the sensor configuration setup module.

The database path is the link parameter for the MySQL local host database. The local database stores all the temperature information in the local server by default. If the user set the remote server address, the received temperature information will also be send to the remote server. The remote server is identified by and IP address and a port number.

The task module manages the sensing data collection for each specific task. It allows several tasks to operate simultaneously without interfering each other. In this way, the software is able to handle multi-task in the same time. So for each task the monitoring parameters and temperature curves will not override with each other.

The communication port number module sets the port for the receiver and configures the serial port which connected to the wireless receiver. All the sensor information submitted will be forwarded to the port so the software can communicate with the wireless sensor network.

The sensor configuration setup sub-module handles all the sensors connected to the software. It can configure the sensor parameters such as the data acquisition interval and the sensor number. For each sensors connected, the software can send command to the sensor to set parameters such as the sensor channel, data acquisition intervals and display the sensor parameters such as the MAC address, the current temperature, the voltage, the sensor name, and the last update time.

### 3.2. Sensors Module

**Figure 13:**
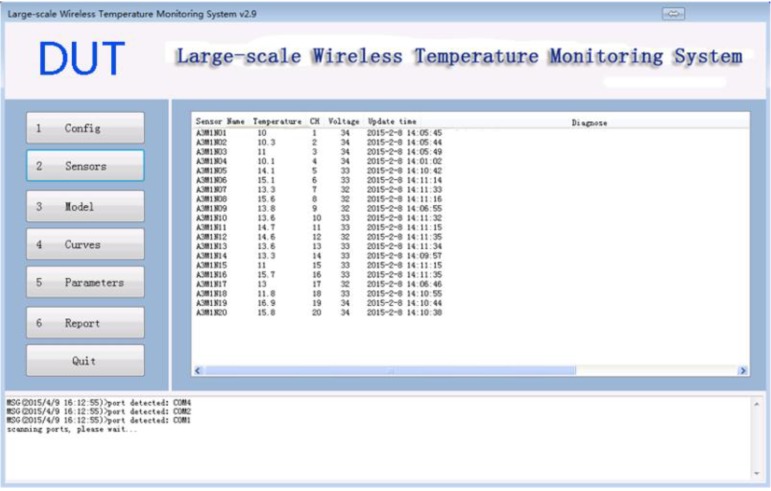
Sensors Module.

The sensors module as shown in Figure 13 displays all the sensors that is currently connected to the software and display the diagnose information such as the sensor name, temperature, voltage, and the last update time. For the self-diagnostic and self-healing purposes, the software checks the health status of each sensor every 10 min. If any error occurs to the sensor, e.g., the sensor is disconnected, the software try to fix it by itself. If the software is not capable of doing so, e.g., the sensor’s battery is running out, the software displays a warning message on the screen. By doing so, the temperature monitoring personnel will be able to fix the errors as quick as possible.

### 3.3. Model Module

**Figure 14:**
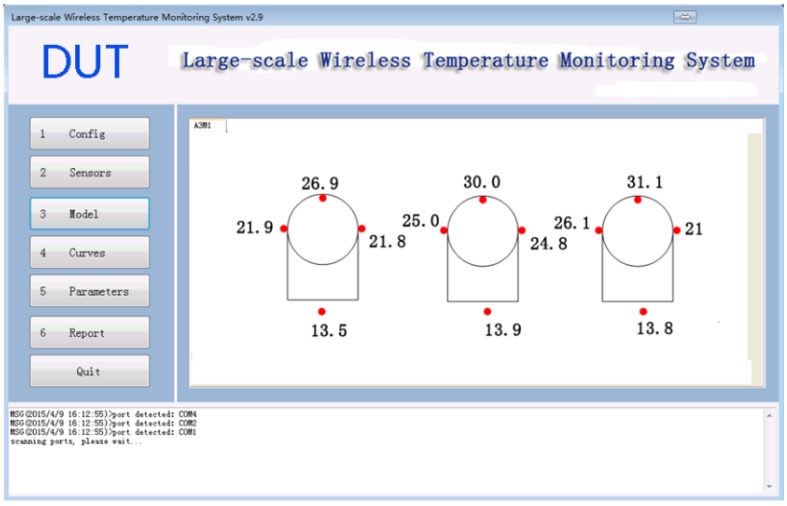
Model Module.

The 3D vision model module as shown in Figure 14 displays all the temperature information on a 3D model. For each monitoring task, an independent tag is generated by the software and for each tag the sensors in the corresponding task will be automatically loaded. This allows the software to monitor several tasks at the same time.

### 3.4. Curves Module

**Figure 15:**
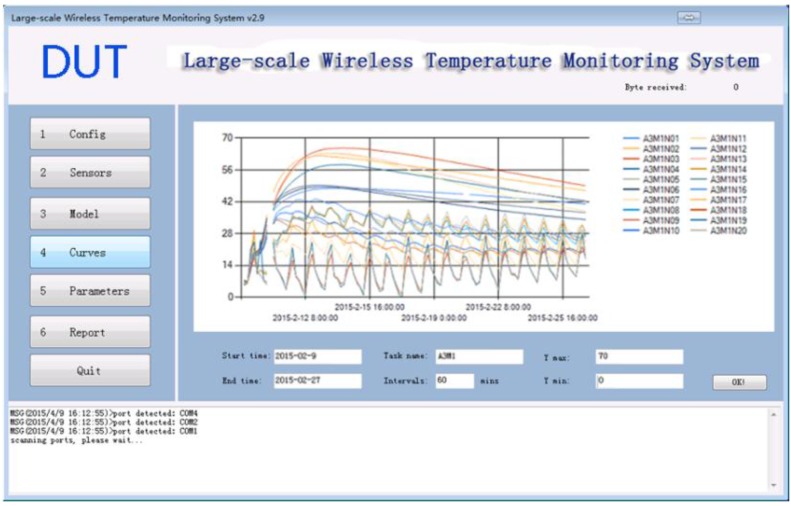
Curve Module.

The temperature curves module shows the curves of all the sensors in one specific task. The user can set the start date and the end date, the task name, the time intervals, and the max/min value for the Y axis, which allows to zoom in and zoom out the curves. Figure 15 shows the history of task which is named as A3M1 from the data obtained from 9 February 2015 to 27 February.

### 3.5. Parameters Module

**Figure 16:**
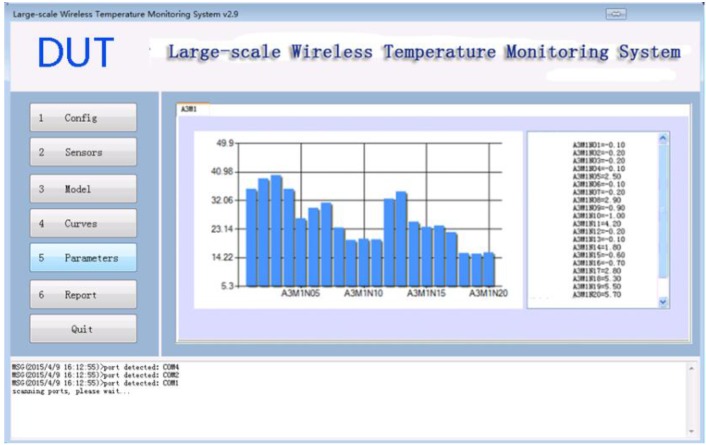
Monitoring Parameters Module.

The monitoring parameter module draws all the sensor temperature information as bar charts as shown in Figure 16. Each bar represents one sensor which allows the user easily notify the maximum and minimum temperature. This module also calculates the temperature changing rate and inner-surface temperature difference according to the history data and the sensor locations.

### 3.6. Data Module

**Figure 17:**
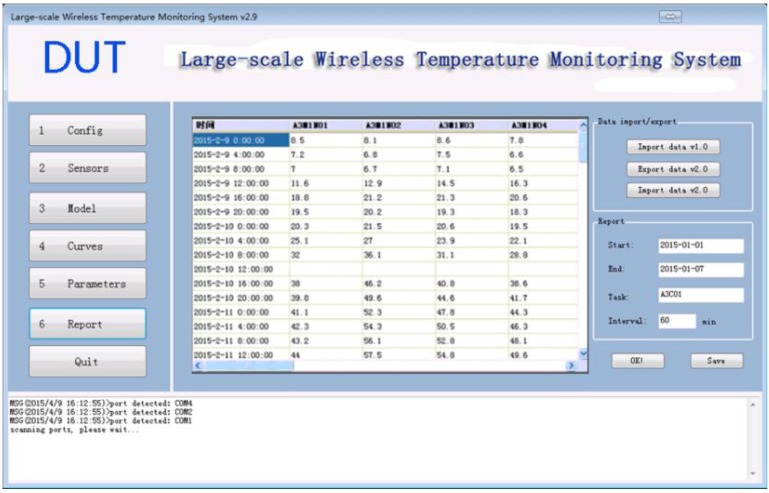
Data Module.

The data module, as shown in Figure 17, imports and exports data. Data import allows data to be transferred via different computers (e.g., to transfer to another backup computer). Data export allows data from the database (which data are stored in binary format) to save as a text file format for other purposes. The user can also retrieve sensor data of a specific monitoring task and display it in a table, as shown in Figure 17. The table can be saved as an Excel spread sheet format for the need of further analysis.

## 4. Case Study of Engineering Application

In order to validate the developed large-scale temperature monitoring system, a case study was investigated to apply the system on real-world LPG storage tanks as described in this section.

### 4.1. Project Overview

**Figure 18:**
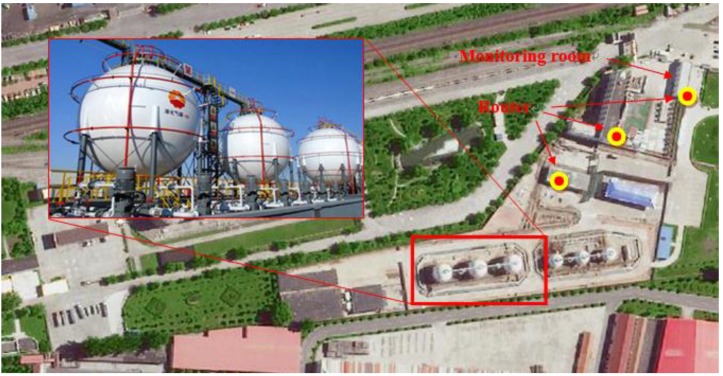
Project overview.

The monitored LPG storage tanks were located at a LPG storage facility in the Shunyi District of Beijing, China, 5 km from the capital airport. There were seven LPG storage tanks on site, including three tanks with a volume of 1000 m^3^, three with 400 m^3^, and one with 50 m^3^, resulting in a total capacity of 4250 m^3^ of LPG in this facility. Figure 18 shows the storage tanks which are of spherical shape. The design pressure of the LPG storage tanks is 2.6 MPa and the design temperature is −15 °C–50 °C.

### 4.2. Deployment of the Monitoring System

**Figure 19:**
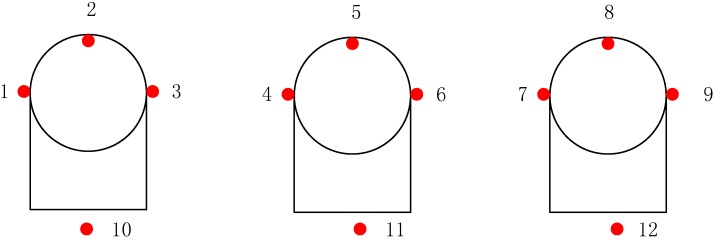
Sensor layout.

To ensure a safe operation of the LPG storage tanks, we have installed nine sensors (sensor numbers 1–9) on three spherical LPG storage tanks as shown in Figure 19 to monitoring the inner temperature, the surface temperature. Three additional sensors (sensor numbers 10–12) were installed to monitor the environment temperature, which can also be seen in Figure 19. The sensor numbered 2, 5, and 8 are fiber optic temperature sensors as described in Section 2.2. The rest of sensors are wireless sensors. The sensor deployment was started on 16 February 2015 and completed on 17 February 2015. The monitoring system was powered on 17 February 2015 and all the sensors survived the installation process. The temperature data was transmitted to the monitoring room via wireless signal. Figure 18 also shows that several routers were used allowing for the wireless signal transmitted from the storage tanks to the monitoring room. The monitoring room was located 1 km away from the storage tank in the office building as shown in Figure 18.

### 4.3. Data Acquisition and Analysis

**Figure 20:**
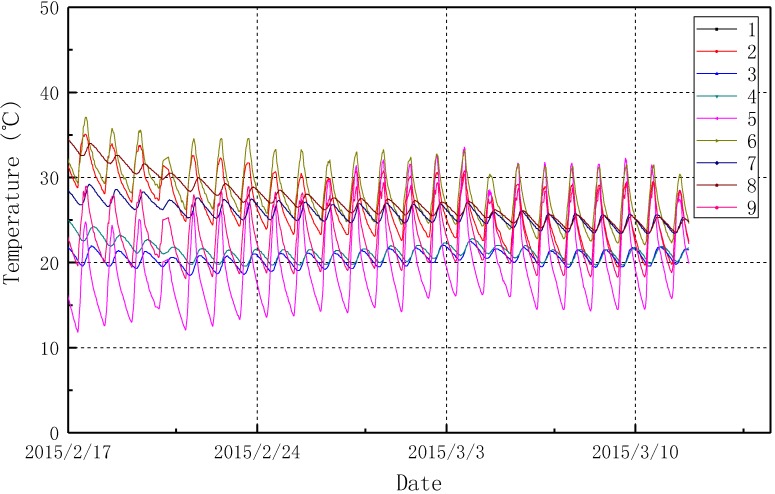
LPG storage tank temperature monitoring data.

**Figure 21:**
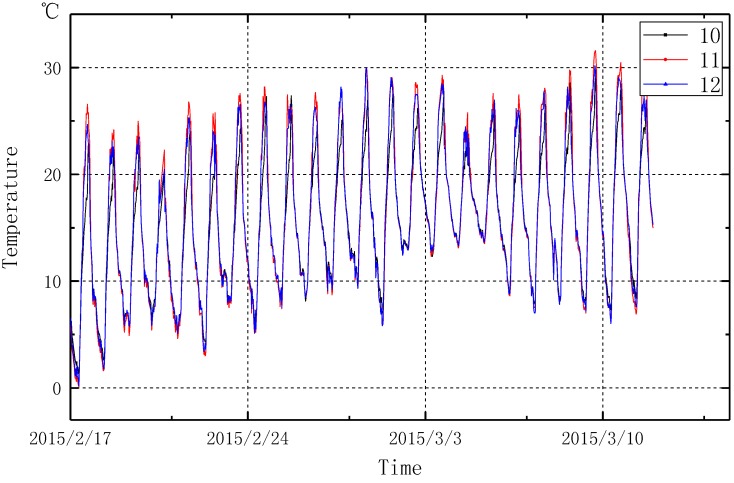
Environment temperature monitoring data.

Figure 20 and Figure 21 show the temperature data monitored from the developed system between 17 February 2015 and 17 March 2015. Figure 21 demonstrated the environmental temperatures monitored from sensor numbers 10–12. During a period of one month, the environment temperature varies significantly up to as much as 20 °C a day. Figure 20 shows the measured temperatures from sensor numbers 1–9 on the tanks. Influenced by the environment temperature changes, the surface temperature and the inner temperature of the LPG storage tanks also have a variation of 10 °C in a day. Within the 30 days of monitoring, the monitoring system shows a good reliability, the monitoring system is expected to operate for one year without maintenance.

## 5. Conclusions

In this paper, a large-scale wireless temperature monitoring system for LPG storage tanks was developed and applied to a real-world monitoring project. The main conclusions are outlined below:

(1) Hardware for the monitoring system was developed which includes wireless temperature sensors, fiber optic high temperature sensors, onsite monitoring system, and the remote access system. The hardware of the monitoring system allows the multi-parameter monitoring, wireless sensing and harsh environments adaptive, local and remote access.

(2) Software for the monitoring system was developed which can configure the sensors’ data acquisition interval, diagnose sensor’s health status, display the monitoring parameters, such as inner temperature, surface temperature, inner-surface temperature difference, and the environment temperature, both in 3D vision and in history curves. The temperature changing rate and the maximum history temperature are calculated by the software automatically. All the data can be exported to Excel file format. The software for the mass concrete fulfills the requirements of automatic monitoring and data management.

(3) The engineering practice validated the feasibility of the developed system and it has advantages, such as being easy to deploy, easy to use, and highly reliability.

Upon validation, the system can be widely applied to the monitoring of LPG storage tanks and other liquid or chemical storage tanks, which will significantly improve the reliability and safety management of these critical facilities.
